# Propagermanium administration for patients with type 2 diabetes and nephropathy: A randomized pilot trial

**DOI:** 10.1002/edm2.159

**Published:** 2020-06-12

**Authors:** Akinori Hara, Miho Shimizu, Erika Hamaguchi, Hirokazu Kakuda, Kenzo Ikeda, Toshiya Okumura, Kiyoki Kitagawa, Yoshitaka Koshino, Motoo Kobayashi, Kazuya Takasawa, Yukimasa Hisada, Tadashi Toyama, Yasunori Iwata, Norihiko Sakai, Takashi Wada

**Affiliations:** ^1^ Division of Nephrology Kanazawa University Hospital Kanazawa Japan; ^2^ Department of Environmental and Preventive Medicine Faculty of Medicine Institute of Medical, Pharmaceutical and Health Sciences Kanazawa University Kanazawa Japan; ^3^ Department of Nephrology and Laboratory Medicine Faculty of Medicine Institute of Medical, Pharmaceutical and Health Sciences Kanazawa University Kanazawa Japan; ^4^ Department of Internal Medicine Japanese Red Cross Kanazawa Hospital Kanazawa Japan; ^5^ Kakuda clinic Kahoku Japan; ^6^ Izumigaoka Medical Clinic Takaoka Japan; ^7^ Department of Internal Medicine Tonami General Hospital Tonami Japan; ^8^ Division of Internal Medicine National Hospital Organization Kanazawa Medical Center Kanazawa Japan; ^9^ Department of Internal Medicine Mizuho hospital Kahoku Japan; ^10^ Department of Internal Medicine Municipal Tsuruga Hospital Tsuruga Japan; ^11^ Department of Nephrology Public Central Hospital of Matto Ishikawa Hakusan Japan

**Keywords:** albuminuria, CCR2, diabetes, kidney, MCP‐1, propagermanium

## Abstract

**Aims:**

We assessed the potential efficacy and safety of propagermanium (PG), an organic compound that inhibits the C–C chemokine receptor type 2, administration in patients with type 2 diabetes and nephropathy. Furthermore, we assessed the feasibility of future studies.

**Materials and methods:**

We recruited patients from nine medical institutions in Japan for this randomized, open‐label, parallel two‐arm pilot trial. Inclusion criteria were diagnosis of type 2 diabetes, age 30‐75 years, dipstick proteinuria of ≥1+ or urinary albumin‐to‐creatinine ratio (UACR) of ≥30 mg/g and estimated glomerular filtration rate of ≥30 mL/min/1.73 m^2^. Patients were randomly assigned (1:2) using a minimization algorithm to either continuing usual care or concomitant administration of 30 mg PG per day for 12 months. The primary outcome was the change in UACR from baseline to 12 months. We also collected safety information for all patients who received at least one dose of PG.

**Results:**

We enrolled 29 patients, 10 were assigned to continue usual care and 19 to receive PG. Changes in UACR by PG in addition to the usual care were 25.0% (95% CI −20.4%, 96.5%, *P* = .33). No severe adverse events or renal events were observed during the study.

**Conclusion:**

Although the treatment with PG was generally well tolerated, the dosage of 30 mg/d for 12 months did not reduce albuminuria when used in addition to usual care in patients with type 2 diabetes and nephropathy. Efficacy of PG should be verified in future definitive trials.

## Graphical abstract

We assessed the potential efficacy and safety of propagermanium (PG), an organic compound that inhibits the C–C chemokine receptor type 2, administration in patients with type 2 diabetes and nephropathy. Changes in albuminuria by PG in addition to the usual care were 25.0% (95% CI −20.4%, 96.5%, *P* = .33). No severe adverse events or renal events were observed during the study.

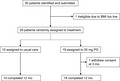

## 1 BACKGROUND

Type 2 diabetes with albuminuria/proteinuria is associated with high rates of renal and cardiovascular morbidity and mortality. Treatments for diabetic nephropathy (diabetic kidney disease), which include controlling blood glucose and blood pressure with angiotensin‐converting enzyme inhibitors or angiotensin receptor blockers, have been proven to be effective and are recommended for reducing renal and cardiovascular risks.^1^ The renoprotective and cardioprotective actions of renin‐angiotensin system (RAS) inhibitors have been attributed, in part, to their albuminuria‐/proteinuria‐lowering effects.^2^ However, some patients remain at risk of the development and progression of renal and cardiovascular diseases, which has been suggested to be related to the presence of high albuminuria/proteinuria.^2^, ^3^, ^4^, ^5^

Treatment options that target other pathways involved in the development and progression of diabetic nephropathy are required for further reducing renal and cardiovascular risk in this patient population. Recent research has revealed, in studies on both patients and animals, monocyte chemoattractant protein (MCP)‐1 (also referred to as C–C chemokine ligand 2) to be important in the progression of diabetic nephropathy and has been indicated the potential of this protein as a marker of renal disease.^6^, ^7^, ^8^, ^9^, ^10^, ^11^, ^12^ Biologically, MCP‐1 plays important roles in chronic inflammatory kidney disease through interaction with its receptor, C–C chemokine receptor type 2 (CCR2), which promotes monocyte and macrophage migration and activation.^13^ Propagermanium (PG; 3‐oxygermylpropionic acid polymer) is an organic compound that has been approved for the treatment of chronic hepatitis B and is currently in use for this purpose in Japan.^14^ It has been reported that PG acts as a CCR2 inhibitor, blocking MCP‐1–dependent monocyte/macrophage activation and chemotaxis.^15^ On the basis of this property, recent studies have investigated the utility of PG as an anti‐inflammatory drug for cardiovascular and renal diseases. Among these, preclinical studies have indicated that PG improves albuminuria/proteinuria in rodent models of diabetes.^16^ Although there is accumulating evidence of the clinical utility of the inhibition of CCR2 by PG, its benefits in human diabetic nephropathy remain to be investigated.

This pilot study aimed to assess the feasibility of conducting a definitive trial of PG as well as to investigate its efficacy and safety for the reduction of albuminuria in patients with type 2 diabetes and nephropathy.

## 2 METHODS

### 2.1 Study design and participants

We recruited patients who were treated at nine medical institutions in Japan between 29 May 2014 and 31 March 2016 for this randomized, open‐label, parallel two‐arm pilot trial. The institutes included outpatient clinics of internal medicine as well as secondary and tertiary hospitals. Researchers at each institution identified potential participants who met the following inclusion criteria: age 30‐75 years, diagnosis of type 2 diabetes ^17^ 5 years or more prior to study participation, dipstick proteinuria ≥ 1+ (corresponding to ≥300 mg/g protein), or urinary albumin‐to‐creatinine ratio (UACR) of ≥30 mg/g, estimated glomerular filtration rate (eGFR; calculated using the equation in the ‘Procedures’ section) of ≥30 mL/min/1.73 m^2^, baseline glycated haemoglobin (HbA1c) of ≤10% and baseline body mass index (BMI) of 19‐40 kg/m^2^.

Exclusion criteria were positive for hepatitis B surface antigen or antibodies to hepatitis C virus; severe liver dysfunction; jaundice; history of arteriosclerosis obliterans, myocardial infarction or stroke within 3 months prior to enrolment; pregnancy; or lactating.

This trial was conducted in accordance with the Declaration of Helsinki. An institutional review board in Kanazawa University Hospital approved the research protocol (No. 5682). All patients provided written informed consent before starting the trial.

### 2.2 Randomization

Participants were randomly assigned to the usual care or PG group using computer‐generated randomization. An unequal randomization of 1:2 was chosen to increase the data relating to PG administration. Following central randomization at the independent data centre, participants were stratified by baseline UACR (30‐300 mg/g and ≥ 300 mg/g) and baseline eGFR (30‐60 mL/min/1.73 m^2^ and ≥ 60 mL/min/1.73 m^2^). The data centre used a minimization algorithm to maintain balance among the two groups with respect to the baseline UACR and eGFR ranges. Participants and all study personnel were not blinded to treatment allocation. We obtained PG from Sanwa Kagaku Kenkyusho Company, Ltd. (Nagoya) supplied as capsules in bottles labelled appropriately for the trial.

### 2.3 Procedures

After randomization, blood and urine specimens were collected at baseline and at the 1, 3, 6, 9, and 12 month visits. Urinary albumin was measured using nephelometric immunoassay, and creatinine was measured using an enzymatic method. Serum creatinine and urea nitrogen were measured at baseline and at 1, 3, 6, 9 and 12 months, whereas urinary MCP‐1 was measured by SRL, Inc (Tokyo, Japan) at baseline and at 12 months using quantitative enzyme‐linked immunosorbent assay and expressed as urinary MCP‐1‐to‐creatinine ratio. Serum creatinine levels were used to calculate eGFR using the following equation for Japanese patients: eGFR (mL/min/1.73 m^2^) = 194 × serum creatinine^−1.094^ × age^−0.287^ (if female, × 0.739).^18^

We measured HbA1c and C‐reactive protein (CRP) at baseline and at 1, 3, 6, 9 and 12 months. For participants assigned to the PG group, plasma concentrations of PG were measured at 1 and 12 months using liquid chromatography‐mass spectrometry in the pharmaceutical technology laboratory of Sanwa Kagaku Kenkyusho Co., Ltd. (Inabe).

Safety was evaluated at baseline and at 1, 3, 6, 9 and 12 months by assessing adverse events and laboratory data. Serious adverse events were defined as any adverse event that resulted in death, was immediately life threatening, required hospital admission, resulted in persistent or substantial disability or incapacity, was a birth defect, or was an important event that might heavily jeopardize the participants or require intervention to prevent any of the above.

We assessed treatment adherence on the basis of interview at each study visit as well as from plasma PG concentrations measured at 1 and 12 months. In order to assess the potential efficacy of PG, changes in the dosage of simultaneously administered drugs including RAS inhibitors, statins and antiplatelet agents were basically not permitted during the study period.

### 2.4 Outcomes

The outcomes of this trial were the number of recruited patients, retention rate and safety of the intervention. The primary efficacy end‐point was the change in UACR from baseline at 12 months. Secondary end‐points included the changes from baseline of eGFR, serum urea nitrogen, HbA1c, total cholesterol, CRP and urinary MCP‐1‐to‐creatinine ratio at 12 months.

### 2.5 Statistical analysis

Outcomes are reported descriptively and narratively. We assessed safety data for all participants who received at least one dose of the study drug. Analyses were based on comparison between the usual care and PG groups and reported as raw count (number, %) for each event.

Since this was a pilot study, a sample size calculation was not performed. We aimed for a total of 30 participants because this was considered reasonable for evaluation of the practicalities of implementing the intervention in daily clinical practice. We performed preliminary efficacy analysis on the intention‐to‐treat population; all assigned participants were included in statistical analysis. The analysis used a mixed‐effects model to estimate the change from baseline to each post‐baseline measurement of log UACR using the statistical package SPSS, version 24 (IBM) or Stata/MP 14.2 (STATA Corp.). The model included treatment, visit and each baseline value as covariates. The UACR values were log‐transformed prior to analysis to reduce data skew. Changes in UACR were calculated as the ratio of the geometric mean of UACR measurements after randomization in the PG group compared to those in the usual treatment, and the same calculation was applied to the 95% confidence intervals (CIs).

Intergroup differences were compared with two‐sided significance level of 0.05. We used similar statistical models to assess differences in other efficacy variables such as eGFR, HbA1c and CRP. The change in urinary MCP‐1‐to‐creatinine ratio from baseline to 12 months was estimated using a multivariable regression model adjusted for baseline MCP‐1.

Concomitant drugs including RAS inhibitors, calcium channel blockers, glucose‐lowering drugs and lipid‐modifying drugs were summarized for each treatment group.

The study was registered with UMIN‐CTR, number UMIN000004779.

### 2.6 Role of the funding source

The study was overseen by the Hokuriku Clinical Research Supporting Center. The data centre of the innovative clinical research centre of Kanazawa University managed and handled all data. The statistical department of the centre was involved in the analysis of data. All authors had access to study results, and the lead author takes responsibility for the accuracy and completeness of the data reported. The lead author had the final decision to submit the publication.

This study was supported by a Grant‐in‐Aid for Scientific Research from the Japan Society for the Promotion of Science (JSPS KAKENHI) Grant number 26893095. Although the study drug was provided by the Sanwa Kagaku Kenkyusho Co., Ltd, the company had no role in the design of the study; collection, analysis, or interpretation of data; writing of the report; or the decision to submit the article for publication.

## 3 RESULTS

### 3.1 Background of the study population

**Figure 1:**
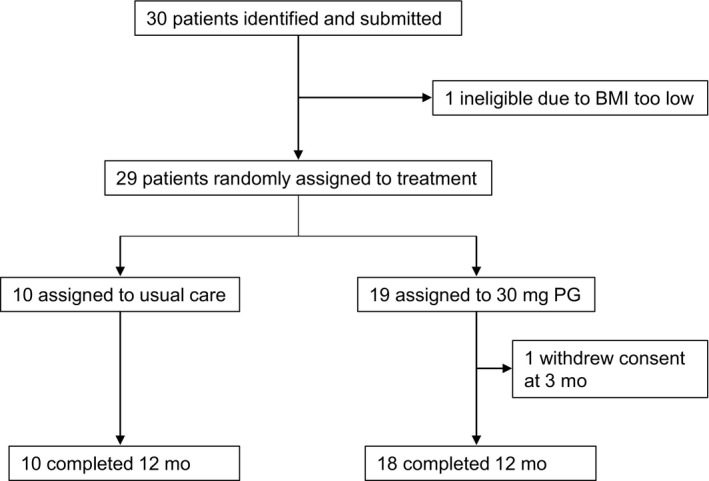
Flow diagram of trial enrolment. Abbreviations: BMI, body mass index; PG, propagermanium

Enrolment began in May 2014 and was completed in March 2016. In total, 30 patients were identified as candidates; of which, 29 were enrolled, and 1 was excluded due to BMI < 19 kg/m^2^. Ten patients were assigned to usual care and 19 to the PG group. One patient assigned to PG group withdrew consent at 3 months because of difficulties accessing our institution. Finally, 28 of the 29 patients completed the total of 12 months of the study (Figure 1).

Baseline demographics, clinical and biochemical characteristics, and concomitant drugs were similar between the groups (Table 1). The median UACR at baseline was not markedly different between the two groups.

**Table 1:** Baseline characteristics of the study population

	Total (n = 29)	Usual care (n = 10)	PG (n = 19)
Demographic and clinical characteristics			
Age (years)	64 (57‐67)	63 (59‐67)	64 (54‐67)
Men	22 (76)	7 (70)	15 (79)
BMI (kg/m^2^)	27.0 (24.6‐28.8)	25.8 (24.2‐28.9)	27.0 (25.3‐28.9)
Known duration of diabetes (years)	15 (8‐20)	16 (11‐20)	13 (8‐20)
Systolic blood pressure (mmHg)	136 (121‐149)	137 (118‐151)	131 (122‐148)
Diastolic blood pressure (mmHg)	78 (73‐85)	79 (67‐86)	78 (76‐84)
White blood cells (×10^3^/mm^3^)	6.50 (5.63‐7.15)	6.35 (5.79‐6.90)	6.61 (5.50‐8.30)
Haemoglobin (g/dL)	13.9 (12.9‐15.4)	13.9 (12.6‐14.6)	13.9 (12.8‐15.6)
Platelet (×10^4^/mm^3^)	23.0 (20.6‐27.4)	24.6 (20.8‐34.0)	22.9 (20.0‐25.5)
Serum urea nitrogen (mg/dL)	14.9 (12.4‐22.2)	21.1 (13.5‐24.8)	13.7 (11.8‐21.3)
Serum creatinine (mg/dL)	0.90 (0.70‐1.25)	0.87 (0.63‐1.50)	0.90 (0.73‐1.14)
eGFR (mL/min/1.73 m^2^)	66.9 (41.1‐82.9)	69.7 (36.8‐91.8)	66.9 (50.7‐75.0)
Uric acid (mg/dL)	6.0 (5.3‐6.8)	5.7 (5.3‐6.7)	6.0 (5.4‐6.8)
AST (U/L)	21 (17‐25)	20 (15‐22)	21 (19‐27)
ALT (U/L)	22 (14‐49)	16 (13‐28)	28 (15‐57)
γGTP (U/L)	37 (23‐47)	33 (20‐56)	40 (24‐48)
Serum albumin (g/dL)	4.4 (4.0‐4.6)	4.5 (4.4‐4.7)	4.3 (3.8‐4.5)
HbA1c (%)	7.1 (6.6‐8.1)	6.8 (6.6‐7.2)	7.6 (6.6‐8.3)
Total cholesterol (mg/dL)	170 (160‐211)	207 (164‐211)	168 (158‐209)
CRP (mg/dL)	0.04 (0.02‐0.18)	0.04 (0.01‐0.06)	0.06 (0.03‐0.35)
UACR (mg/gCr)	229 (105‐747)	470 (106‐690)	192 (55‐1078)
Urinary MCP‐1‐to‐creatinine ratio (pg/mg)	285.3 (200.0‐482.8)	304.8 (240.4‐487.8)	280.3 (197.4‐499.8)
Drug treatment			
Drugs used in diabetes			
Biguanides	8 (28)	3 (30)	5 (26)
α‐glucosidase inhibitors	5 (17)	3 (30)	2 (11)
Thiazolidinediones	9 (31)	4 (40)	5 (26)
Sulfonylureas	9 (31)	1 (10)	8 (42)
Insulin and its analogs	8 (28)	4 (40)	4 (21)
DPP4 inhibitors	23 (79)	9 (90)	14 (74)
SGLT2 inhibitors	2 (7)	0	2 (11)
Statins	22 (76)	6 (60)	16 (84)
Antihypertensives			
ACE inhibitors	3 (10)	1 (10)	2 (11)
Angiotensin receptor blockers	21 (72)	8 (80)	13 (68)
Calcium channel blockers	22 (76)	8 (80)	14 (74)
Others^a^	2 (7)	2 (20)	0
Drugs used in cardiovascular diseases			
Antiplatelets	7 (24)	4 (40)	3 (16)

Note

Data are expressed as median (interquartile range) or n (%).

Abbreviations: ALT, alanine aminotransferase; and ACE, angiotensin‐converting enzyme; AST, aspartate aminotransferase; BMI, body mass index; CRP, C‐reactive protein; DPP4, dipeptidyl peptidase 4; eGFR, estimated glomerular filtration rate; HbA1c, glycated haemoglobin; MCP‐1, monocyte chemoattractant protein‐1; PG, propagermanium; SGLT2, sodium glucose cotransporter 2; UACR, urinary albumin‐to‐creatinine ratio; γGLT, gamma glutamyl transpeptidase.

^a^ Others include an alpha blocker and a thiazide diuretic.

### 3.2 Change in albuminuria

**Figure 2:**
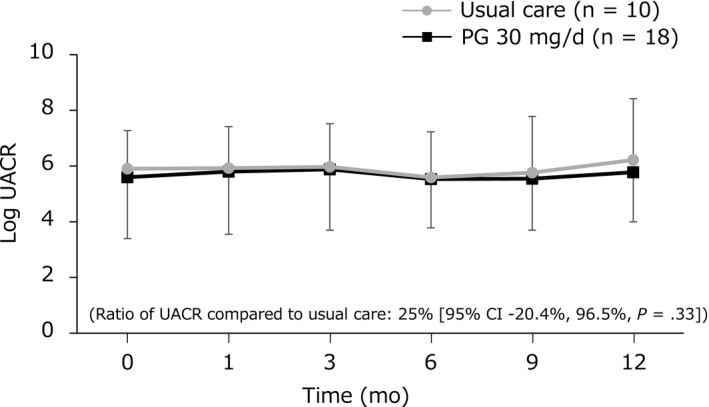
Time course of albuminuria. Abbreviations: CI, confidence interval; PG, propagermanium; UACR, urinary albumin‐to‐creatinine ratio

Figure 2 shows the course of log UACR over the 12 months. Changes in UACR by PG in addition to the usual care were 25.0% (95% CI −20.4%, 96.5%) and were not significantly different (*P* = .33).

### 3.3 Other outcomes

Changes in eGFR, HbA1c, serum urea nitrogen, CRP, serum cholesterol and urinary MCP‐1 were not statistically different between the two groups (Table 2).

**Table 2:** Change from baseline to 12 months according to treatment group

	Usual care		Propagermanium		*P* value vs placebo
	n	Least‐squares mean (95%CI)	n	Least‐squares mean (95%CI)	
eGFR (mL/min/1.73 m^2^)	10	2.17 (−3.22, 7.56)	19	−0.70 (−4.63, 3.24)	.41
HbA1c (%)	10	0.18 (−0.05, 0.42)	19	0.23 (0.06, 0.40)	.76
Serum urea nitrogen (mg/dL)	10	−0.50 (−3.02, 2.03)	19	0.41 (−1.43, 2.24)	.58
T‐Chol. (mg/dL)	10	−0.64 (−15.95, 14.68)	18	8.78 (−2.72, 20.29)	.35
CRP (mg/dL)	10	−0.20 (−0.29, −0.11)	17	−0.12 (−0.19, −0.05)	.21
Urine MCP‐1 (pg/mgCr)	9	70.56 (−178.4, 319.5)	16	183.1 (−3.2, 369.5)	.46

Note

Data are expressed as least‐squares mean (95% confidence intervals).

Abbreviations: 95%CI, 95% confidence interval; CRP, C‐reactive protein; eGFR, estimated glomerular filtration rate; MCP‐1, monocyte chemoattractant protein‐1; T‐Chol., total cholesterol; UACR, urinary albumin‐to‐creatinine ratio.

### 3.4 Safety

The study drug was generally well tolerated (Table 3). Adverse events were consistent with age and underlying medical conditions of the study population. Serious adverse events including end‐stage renal failure requiring dialysis therapy and death were not observed during the study period in PG group.

**Table 3:** Adverse events in the study population

	Usual care (n = 10)	PG (n = 19)
Quick to fatigue	0	1 (5)
Diarrhoea	0	1 (5)
Peripheral numbness	0	1 (5)
Dizziness	1 (10)	0
Oedema of lower legs	0	2 (11)
Varices of lower legs	1 (10)	0
Foot ulcer	0	1 (5)
Cough	1 (10)	0
Upper respiratory infection	3 (30)	3 (16)
Iron deficiency anaemia	0	1 (5)

Note

Data are presented as n (%).

Abbreviation: PG, propagermanium.

With regard to treatment adherence, the median plasma concentrations of PG at 1 and 12 months after starting the drug were 8.73 ng/mL (0‐47.80) and 14.10 ng/mL (0‐52.88), respectively. In contrast, although 19 patients assigned to the PG group declared their adherence at each visit, plasma PG concentrations at both 1 and 12 months were lower than the limit of detection (5.00 ng/mL) in three participants.

## 4 DISCUSSION

Although we found PG to be generally well tolerated by patients with type 2 diabetes with nephropathy and severe adverse events were not observed, a dosage of 30 mg/d did not decrease albuminuria over the 12 months of the study. To the best of our knowledge, this is the first clinical trial to use PG in patients with diabetic nephropathy.

Recent advances in the understanding of the pathophysiology of diabetic nephropathy have focused on inflammatory pathways as therapeutic targets.^13^, ^19^, ^20^ Among these, the MCP‐1/CCR2 pathway has attracted particular interest because this pathway has been demonstrated to be associated with renal function decline. Furthermore, inhibition of MCP‐1 and CCR2 has been shown to ameliorate renal function and pathological development in experimental models of diabetic nephropathy.^13^, ^16^ The anti‐inflammatory compound PG was developed and has been authorized for use in Japan. Clinically, this drug has been used for the treatment of chronic hepatitis B, and its mechanism of action is known to involve activation of cytotoxic T cells and NK cells, leading to the destruction of cells infected with the hepatitis B virus.^14^ As a secondary effect, the inhibition of MCP‐1/CCR2 by PG has been reported recently.^15^ Since then, the efficacy of PG for the treatment of inflammation of the kidney, liver and brain ^21^, ^22^, ^23^, ^24^, ^25^; diabetes ^26^, ^27^; and nephropathy^16^ has been reported in animal models. On the basis of these biological effects, this pilot study assessed the feasibility and potential efficacy of CCR2 inhibition by PG in terms of albuminuria as well as HbA1c and CRP levels in patients with type 2 diabetes and nephropathy.

In the present study, there was no difference in the change in albuminuria between participants who received PG and those who received usual care. Other than the small sample size of this pilot study, this observation may be explained by the fact that, although the standard dose of PG used for adult patients with chronic hepatitis B is generally 30 mg/d,^14^ this dose may be insufficient to inhibit CCR2 in the context of diabetes in humans. Treatment adherence of participants assigned to the PG group may not have been sufficient, as indicated by plasma PG concentrations, which were lower than the limit of detection for some patients. Participants assigned to the usual care group exhibited good self‐management and treatment adherence, as indicated by baseline characteristics such as BMI and HbA1c (Table 1).

During the study period, the results of related randomized clinical trials performed in European countries and the United States were reported. One trial reported that CCX140‐B induced a significant reduction in albuminuria of between 10% and 16% when used concomitantly with RAS inhibitors.^28^ This effect persisted for 4 weeks after cessation of administration, suggesting nonhemodynamic effects.^28^ Another trial was involving administration of a dual CCR2/5 antagonist, PF‐04634817, to patients with macroalbuminuria demonstrating a placebo‐adjusted reduction in UACR of 8.2% at week 12.^29^ In contrast, SGLT2 inhibitors and GLP‐1 receptor agonists have been recommended for the treatment of diabetic nephropathy in daily clinical settings.^1^ These drugs are known to have antihypertensive and antialbuminuric effects, through mechanisms that appear to be independent of glycaemia.^1^ Among those in the present study, the number of participants who used this class of drugs was relatively small. These findings suggest that, if SGLT2 inhibitors and GLP‐1 receptor agonists were more widely used, the requirement for CCR2 inhibitors may change. Therefore, the collection of more clinical information and identification of biomarkers to predict benefit from CCR2 inhibitors in addition to current standard of care may be warranted.

In this study, there was no difference in HbA1c level, a secondary end‐point, between the PG group and usual care groups. A previous randomized trial involving patients with type 2 diabetes reported that administration of a CCR2 antagonist, JNJ‐41443532, resulted in decreased levels of weighted‐mean and fasting plasma glucose over 28 days of treatment.^30^ However, although the homeostasis model assessment of insulin resistance (HOMA‐IR) tended to decrease, the change from baseline to day 28 was not statistically significant. Another trial involving patients with type 2 diabetes and nephropathy reported that changes in HbA1c, fasting plasma insulin and HOMA‐IR in response to a CCR2 inhibitor, CCX140‐B, were not significant compared with the response to the placebo.^28^ Taken together, these data suggest that CCR2 inhibitors might be beneficial for patients with glycaemia and insulin resistance, but further studies are required, as has been indicated previously.^24^, ^31^

We found the 30 mg dosage of PG to be generally well tolerated when administered for 12 months, and no clinically meaningful treatment‐related vital signs data or laboratory abnormalities including liver function test were observed. The PG concentrations at 1 and 12 months were within the range reported in previous studies.^32^ In the current datasheet, the supplier has reported that caution should be taken in as the use of PG in patients with chronic hepatitis B may cause worsening of hepatitis.^32^ This may become an issue for future definitive trials because patients with chronic infectious diseases including not only hepatitis B virus infection but also tuberculosis, which are still prevalent especially in Asia, will have to be excluded.

The present study has some limitations that should be acknowledged. First, we did not assess the number of patients who were initially screened at each institution. This prevents us from assessing the ease of recruitment and thus the potential enrolment for future trials. Second, this is a pilot study with small sample size examining the feasibility of using PG to treat diabetic nephropathy in patients with type 2 diabetes; we cannot evaluate the efficacy of PG with sufficient statistical power. Third, the dosage of PG was 30 mg/d only; the appropriate dose of PG for diabetic nephropathy remains unknown. Fourth, evaluation of treatment adherence for PG may have been insufficient. Although we interviewed participants at each visit and measured the serum concentration of PG at 1 and 12 months, we have no direct and convincing evidence of CCR2 inhibition at the systemic and renal levels. Although we evaluated urinary MCP‐1 concentration as a candidate biomarker of the severity of diabetic nephropathy,^12^ monitoring of pharmacological inhibition of CCR2 may be required to further investigate the efficacy of PG in future definitive trials. Despite these limitations, this pilot study provides suggestive evidence that PG can be safely administered to patients with type 2 diabetes and nephropathy under certain conditions.

In summary, we demonstrate that the administration of PG as a treatment for type 2 diabetes with nephropathy is generally tolerated, although a dose of 30 mg/d for 12 months does not reduce albuminuria when used in addition to usual care. The use of PG as a therapeutic modality for diabetic nephropathy as well as type 2 diabetes requires further investigation, which may elucidate the potential role of CCR2.

## CONFLICT OF INTERESTS

The authors have no conflicts of interest to declare.

## AUTHORS’ CONTRIBUTIONS

AH and TW designed the study. AH, MS, EH, HK, KI, TO, KK, YK, MK, KT, YH and NS enrolled patients. TT and YI performed data analysis and statistical analysis. All authors interpreted the data. AH wrote the original draft of the report, and all authors contributed to its revisions. AH takes responsibility for the full report.

## ETHICAL APPROVAL

This trial was conducted in accordance with the Declaration of Helsinki. An institutional review board in Kanazawa University Hospital approved the research protocol (No. 5682). All patients provided written informed consent before starting the trial. The study was registered with UMIN‐CTR, number UMIN000004779.

## Data Availability

The data that support the findings of this study are available from the corresponding author, upon reasonable request.
